# Improved Photodynamic Efficacy of Zn(II) Phthalocyanines via Glycerol Substitution

**DOI:** 10.1371/journal.pone.0097894

**Published:** 2014-05-19

**Authors:** Yunni Chin, Siang Hui Lim, Yunus Zorlu, Vefa Ahsen, Lik Voon Kiew, Lip Yong Chung, Fabienne Dumoulin, Hong Boon Lee

**Affiliations:** 1 Department of Pharmacology, Faculty of Medicine, University of Malaya, Kuala Lumpur, Malaysia; 2 Department of Pharmacy, Faculty of Medicine, University of Malaya, Kuala Lumpur, Malaysia; 3 Drug Discovery Laboratory, Cancer Research Initiatives Foundation (CARIF), Subang Jaya, Selangor, Malaysia; 4 Department of Chemistry, Gebze Institute of Technology, Gebze, Kocaeli, Turkey; MGH, MMS, United States of America

## Abstract

Phthalocyanines are excellent photosensitizers for photodynamic therapy as they have strong absorbance in the near infra-red region which is most relevant for *in vivo* activation in deeper tissular regions. However, most phthalocyanines present two major challenges, ie, a strong tendency to aggregate and low water-solubility, limiting their effective usage clinically. In the present study, we evaluated the potential enhancement capability of glycerol substitution on the photodynamic properties of zinc (II) phthalocyanines (ZnPc). Three glycerol substituted ZnPc, **1–3**, (tetra peripherally, tetra non-peripherally and mono iodinated tri non-peripherally respectively) were evaluated in terms of their spectroscopic properties, rate of singlet oxygen generation, partition coefficient (log *P*), intracellular uptake, photo-induced cytotoxicity and vascular occlusion efficiency. Tetrasulfonated ZnPc (ZnPcS_4_) was included as a reference compound. Here, we showed that **1–3** exhibited 10–100 nm red-shifted absorption peaks with higher molar absorptivity, and at least two-fold greater singlet oxygen generation rates compared to ZnPcS_4_. Meanwhile, phthalocyanines **1** and **2** showed more hydrophilic log *P* values than **3** consistent with the number of glycerol attachments but **3** was most readily taken up by cells compared to the rest. Both phthalocyanines **2** and **3** exhibited potent phototoxicity against MCF-7, HCT-116 and HSC-2 cancer cell-lines with IC_50_ ranging 2.8–3.2 µM and 0.04–0.06 µM respectively, while **1** and ZnPcS_4_ (up to 100 µM) failed to yield determinable IC_50_ values. In terms of vascular occlusion efficiency, phthalocyanine **3** showed better effects than **2** by causing total occlusion of vessels with diameter <70 µm of the chorioallantoic membrane. Meanwhile, no detectable vascular occlusion was observed for ZnPcS_4_ with treatment under similar experimental conditions. These findings provide evidence that glycerol substitution, in particular in structures **2** and **3**, is able to improve the photodynamic properties of ZnPc.

## Introduction

Photodynamic therapy (PDT) is an effective modality mainly approved for the palliative and curative treatment of some forms of cancers and precancerous lesions. In PDT treatment, non-toxic photosensitizers are administered topically or systematically into the body, and are allowed to passively accumulate at the tumor site. Subsequent irradiation of the lesion tissue with harmless visible light of specific wavelength activates the photosensitizers to produce cytotoxic singlet oxygen species from molecular oxygen. Due to the short half-life of singlet oxygen of approximately 3 µs in cells, damage cause by the singlet oxygen are highly localized, causing irreversible damage to cancerous tissue while sparing the surrounding healthy tissues [1], [2]. To date, most of the photosensitizers used in PDT studies, namely porphyrins, chlorins, pyropheophorbides and hypocrellins, are activated by light of shorter wavelengths. The drawback of light with shorter wavelength is the limitation of their tissue penetration depth which fails to activate photosensitizers accumulated in bulky or deep-seated tumor lesions. Instead, they may activate photosensitizers that are distributed non-specifically in the skin, leading to undesired skin photosensitivity [3], [4], [5], [6], [7], [8]. In order to overcome this problem, photosensitizers with far-red absorption wavelength have been proposed for use in PDT as far-red excitation light has better tissue penetration depth.

Phthalocyanine is one class of chromophore currently being actively investigated as potential photosensitizers for PDT. They are excellent candidates as they absorb strongly in the red and near infrared (NIR) regions of the visible spectrum which correspond to the tissue optical window, thereby allowing activation of photosensitizers within deeper tissue regions. In addition, their minimal absorption at 400–600 nm would minimize the effects of skin photosensitization caused by sunlight. Phthalocyanines also have high chemical- and photo-stability. However, their use in clinical PDT is limited by their poor water solubility and strong tendency to form aggregates [9], [10]. These limitations of phthalocyanines may be overcome by structural modifications, for example, addition of central metal/atom such as Zn^2+^, Al^3+^ and Si^4+^; or attachment of functional groups at the peripheral and non-peripheral positions of the phthalocyanine core. Such modifications may improve their NIR absorption characteristics, water-solubility and pharmacokinetic behavior, making them more suitable for PDT [10], [11]. So far, most studies that attach functional groups to phthalocyanines to improve their water solubility have focused on the synthesis of sulfonated phthalocyanine. However, sulfonation had resulted in the reduction of singlet oxygen generation [12], [13]. Therefore, functionalization of phthalocyanine with other hydrophilic moieties to improve its aqueous solubility while maintaining its biological activity is needed.

**Figure 1:**
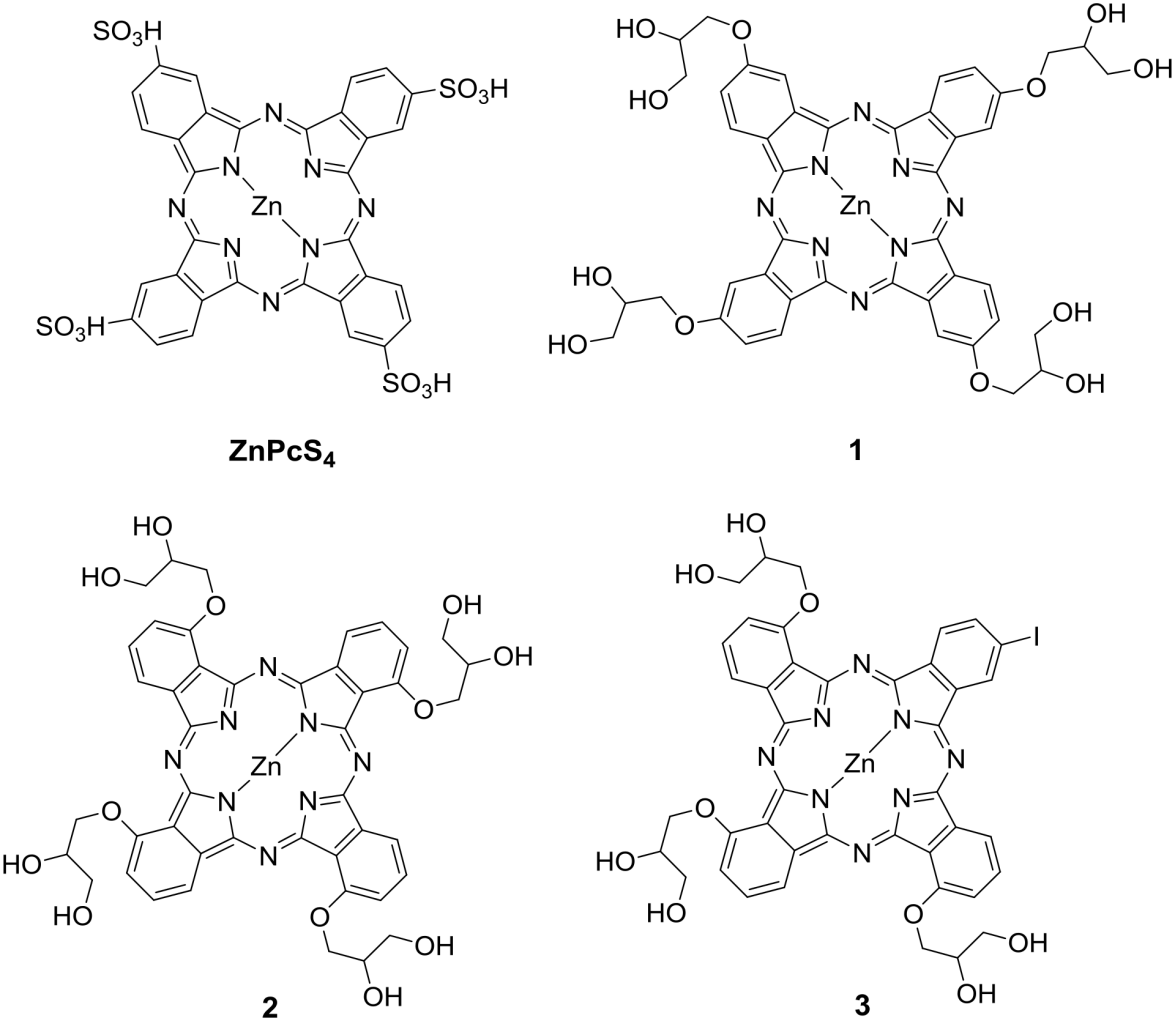
Structures of modified zinc (II) phthalocyanines. Structures of zinc (II) phthalocyanines that were evaluated in this study. Modification of zinc (II) phthalocyanines **1**, **2**, and **3** were done and reported in [11], [22]. ZnPcS_4_ (tetrasulfonated zinc (II) phthalocyanine) was used as a standard. Only one of the possible regioisomers of phthalocyanines **1** (tetra-glycerol peripheral substitution), **2** (tetra-glycerol non-peripheral substitution) and **3** (mono-iodo peripheral and tri-glycerol-non-peripheral substitution) is shown here.

In this present study, three Zn(II) phthalocyanines (ZnPc), namely **1**, **2** and **3** with different glycerol-based substitution pattern were selected for comparative investigations in terms of *in *vitro PDT efficacies and vascular occlusion efficiencies in chick embryo chorioallantoic membrane (CAM) model. The structures of these phthalocyanines are shown in Figure 1, where **1** was tetrasubstituted with glycerol groups at peripheral positions, **2** was tetrasubstituted at non-peripheral positions, and **3** had one peripheral iodine atom facing three glycerol units in non-peripheral positions. All phthalocyanines **1**, **2** and **3** consisted of a mixture of positional isomers. Tetrasulfonated phthalocyanine (ZnPcS_4_), which is commercially available and water-soluble was included as control. This study did not include, the non-substituted ZnPc as a reference because it has limited solubility even in organic solvents. The use of glycerol as the substituent in this study was expected to increase their aqueous solubility while reducing the formation of aggregates. In addition, the attachment of electron donating groups such as alkoxy (i.e. glycerol) to the phthalocyanine macrocycle are also known to increase and red-shift their absorption maxima [10]. Meanwhile, non-peripheral substitution in **2**, compared to the peripheral substitution in **1**, was expected to reduce aggregation and to shift the absorption spectrum towards NIR [11], [12], [14] while, **3** was designed with an iodine atom added to enhance intersystem crossing (ISC) efficiency and singlet oxygen quantum yield [15], [16], [17], [18], [19], [20].

## Experimental Section

### Materials

Cell culture reagents including MEM medium, foetal bovine serum (FBS), penicillin-streptomycin, and trypsin were purchased from Gibco, Invitrogen (USA). 1,3-diphenylisobenzofuran (DPBF), isopropanol, Octanol-1, ethanol, PBS, Triton X-100, and polyethylene glycol 400 (PEG 400) were from Fisher Scientific (Malaysia). Cremophor-EL (CrEL), RPMI 1640 medium, dimethyl sulfoxide (DMSO), and fluorescein isothiocyanate dextran (FITC-dextran, 20 kD), were purchased from Sigma-Aldrich (USA). Thiazoyl blue tetrazolium bromide (MTT) was purchased from Amresco (USA).

### Photosensitizers

The three glycerol-substituted phthalocyanines **1**, **2**, and **3** depicted in Fig. 1 were synthesized as previously reported [21], [22]. NMR and mass spectra of **1**, **2** and **3** were consistent with previous studies [11], [22]. ZnPcS_4_ was purchased from Frontier Scientific Inc. (Utah, USA).

### Photophysical and Photochemical Properties

The UV-visible (10 µM) and fluorescence emission spectra (5 µM) of phthalocyanines in different solvents were measured using SpectraMax M4 microplate reader (Molecular Devices, CA). UV-visible spectra were collected for wavelengths ranging from 350–800 nm and molar extinction coefficient (ε) for each compound was determined. Meanwhile, the fluorescence emission spectra of the compounds were recorded at 650–800 nm following excitation at their respective maximal absorption (λ_max_). The fluorescence quantum yields (φ_F_) were calculated by using the following formula: φ_F_ = φ_Fstd_ (F×A_std_)/(F_std_×A_std_) where, F and F_std_ refer to the area under the fluorescence curve of sample and standard, respectively. A and A_std_ refer to the absorbance of sample and standard compound at their excitation wavelengths respectively. ZnPcS_4_ with φ_Fstd_ = 0.07 (DMSO) was used as reference [13].

### Comparative Singlet Oxygen Generation Rate Measurement

Experiments to measure singlet oxygen generation were carried out as previously described [23]. Briefly, 8 ml solution of aerated isopropanol containing 50 µM of 1,3-diphenylisobenzofuran (DPBF) and 0.5 µM of phthalocyanine or pheophorbide *a* (Pha) in a 6-well plate was irradiated at 5 mW/cm^2^ with light filtered with Roscolux “Light Red” filter no. 22 (>580 nm) (Rosco, NY) at room temperature for 15 min. Aliquots of 200 µl were removed from the mixture and transferred into a 96-well plate at various time intervals. The absorbance of DPBF was measured at 410 nm with a microplate reader. The rate of singlet oxygen production was determined from the reduction of DPBF absorbance over time. Irradiation of DPBF-isopropanol solution in the absence of photosensitizers was also carried out as negative control. The singlet oxygen generation rate for each phthalocyanine was determined relative to Pha which has a singlet oxygen quantum yield of 0.52 in alcohol [24].

### Log P-Partition Coefficient Study

Phthalocyanines were prepared in presaturated Octanol-1 (Oct) or H_2_O depending on their solubility (ZnPcS_4_, phthalocyanines **1** and **2** in H_2_O; phthalocyanine **3** in Oct) with concentrations ranging from 1–20 µM. The same ratio of the other solvent (Oct or H_2_O) was added, vortexed vigorously in a glass tube and centrifuged at 1000 rpm for 2 min. The glass tube was left to stand for 1–2 h at room temperature. The absorbance of the phthalocyanine was measured before addition of the other solvent and also after incubation. To obtain log *P* value, calculations were done using the formula: log *P* = log [C_Oct_/C_H2O_] where, C_Oct_ and C_H2O_ represent concentrations of phthalocyanine in Oct and H_2_O, respectively.

### Cellular Uptake Study

HSC-2 cells were plated overnight in 6-well plates (1×10^5^ cells/well) in MEM media. Cells were incubated with 10 µM of phthalocyanine **1**, **2**, **3** or ZnPcS_4_ for various time intervals. Following incubation, cells were washed twice with PBS, collected by trypsinization and resuspended in phenol red-free MEM media. The mean fluorescence signal for 10 000 cells were analyzed using FACSCalibur flow cytometer (Becton Dickinson, NJ) with FL4 channel (excitation 635 nm, bandpass filter 661/16 nm) and data collected were translated into relative concentration using the formula F = k * QE * P_o_ * (2.303 *ε * b * c). All assays were performed in triplicates [25]. F = measured fluorescence intensity, k = geometric instrumental factor, QE = quantum efficiency (ratio of photons being emitted to photons being absorbed) and Po = radiant power of the excitation source (wavelength-dependent molar absorptivity coefficient). b and c are as being used in the Beer-Lambert law, where b = path length, and c = concentration of the analyte.

### *In vitro* Phototoxicity Assay

MCF-7 human breast carcinoma and HCT-116 cells human colon carcinoma cell lines were obtained from American Tissue Culture Collection (Virginia, USA), whereas HSC-2 human oral squamous carcinoma cell line was purchased from Health Science Research Resources Bank, (Osaka, Japan). Approximately 3000–4000 cells/well were seeded in 96-well plate and incubated overnight for cell adherence and growth. Cells were then treated with phthalocyanines **1**, **2**, **3** or ZnPcS_4_ with concentrations ranging from 0.01 to 100 µM and were incubated for 2 h before irradiated with 4.0 J/cm^2^ of light filtered with Roscolux #26 Light Red (>580 nm) (Rosco, NY) membrane filter at a fluence rate of 7.5 mW/cm^2^ for 10 min. Illumination was performed in the presence of photosensitizer in the media. The cells were further incubated for 48 h before cell viability was assessed by the MTT assay. For MTT assay, 20 µl of MTT solution (5 mg/ml) were added to each well and incubated for 4 h. The medium was removed and replaced with 100 µl of DMSO to dissolve the formazan crystal formed and the absorbance was read at 570 nm with a microplate reader. The percentage of cell viability was determined relative to untreated control and the half maximal inhibitory concentration (IC_50_) values were determined. Dark toxicity for each phthalocyanine was also determined in parallel.

### Statistics

Statistical analysis was performed using GraphPad Prism, version 5.0 (GraphPad Software Inc. San Diego, CA). Quantitative data were expressed as mean ± standard deviation (SD) from at least three independent experiments and assessed using a one-way ANOVA *post-hoc* with Bonferroni test. Differences were considered statistically significant when p<0.05.

### PDT on the CAM Vasculature

Freshly fertilized eggs from Lohmann Brown chicken variety (Hong Hing Sdn. Bhd, Selangor, Malaysia) were incubated with the narrow apex down in a 90° swinging Octagon 40DX incubator (Brinsea Products Inc., Standford, England) at 37°C and 65% relative humidity. On embryo development day (EDD) three, an eggshell opening of about 4 mm in diameter was bored at the apex and sealed with adhesive tape to avoid contamination and desiccation of the egg contents. The eggs were further incubated in stationary position with the apex upright until ready for use at EDD-9.

An early compound formulation was assessed using the CAM model at EDD-9. Two surfactants namely polyethylene glycol 400 (PEG 400) and Cremophore-EL (CrEL) were studied as excipients for these phthalocyanines. Each of the formulations was injected intravenously at the main vasculature of the CAM, using a microliter syringe with 33 gauge needle (Hamilton, Reno NV). The local tolerability of injection such as vasculature disruption, drug precipitation, and 24 h embryo survival were monitored.

For vascular occlusion study, the egg opening was extended to ∼30 mm in diameter on EDD-9. Embryos were intravenously administered with a single bolus of 10 nmol/embryo of phthalocyanine in dosing vehicle (5% CrEL, 5% EtOH in saline) at the CAM main vasculature. One min after injection, a site with blood vessel diameter between 5–100 µm was irradiated with a light dose of 5 J/cm^2^ or 10 J/cm^2^. Irradiation was performed with light of 600–800 nm wavelength with a fluence rate of 11.5 mW/cm^2^ over an irradiation area of 1.13 mm^2^. Fluorescence angiograms were performed immediately to assess the PDT-induced vasculature damage. Blood vessels were perfused with 20 µl of 25 mg/ml FITC-dextran followed by injection of Indian ink into the extra-embryonic cavity to decrease the embryo’s interfering fluorescence from deeper located vessels. The vasculature network at the site of irradiation was illuminated by exciting FITC at 465–496 nm wavelengths on the fluorescence microscope. The vasculature network was imaged and the extent of damage induced by PDT was scored using a previously defined arbitrary damage scale from 1 to 5 as listed in Table 1 [26]. Generally, the damage on smaller vessels would have lower score whereas the damage on larger blood vessels would have higher score, depending on the size and degree of damage that was caused. At least 10 embryos were assessed for each treatment group.

**Table 1:** Damage score of PDT-induced vasculature network occlusion [26].

Occlusion Score	Findings
0	No occlusion
1	Partial closure of capillariesof diameter <10 µm
2	Closure of capillary system, partial closure ofblood vessel of diameter of <30 µm, and size reductionof larger blood vessels
3	Closure of vessels of diameter of <30 µm and partialclosure of larger blood vessels
4	Total closure of vessels of diameter of <70 µm andpartial closure of larger vessels
5	Total occlusion of vessels in the irradiated area

## Results and Discussion

### Photophysical Properties

**Figure 2:**
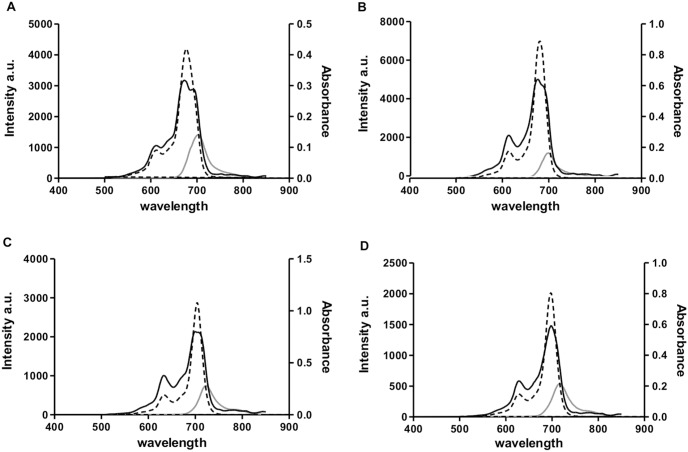
Absorption, emission and excitation spectra of zinc (II) phthalocaynines. Absorption, emission and excitation spectra of (A) ZnPcS_4_, (B) Phthalocyanine **1**, (C) Phthalocyanine **2**, and (D) Phthalocyanine **3** (**—** Emission; **—** Excitation; ---Absorption) measured in DMSO. The excitation and absorption spectra were similar for all phthalocyanines.

All glycerol-substituted phthalocyanines (**1**, **2**, and **3**) were observed to have red-shifted lambda maxima with higher molar extinction coefficient values as compared to ZnPcS_4_ (Table 2 and Figure 2). These characteristics may allow for the use of light with longer wavelengths that can penetrate farther into the tissue, thereby enabling more effective treatment of deep-seated lesions [27]. Attachment of electron donating groups such as NH_2_, OR and SR, either at the non-peripheral or peripheral positions of the phthalocyanine ring has been previously reported to red-shift the absorption to NIR region [28]. Our results concur with these findings and suggest that glycerols, as electron-donating alkoxy groups, were responsible for the observed bathochromic shifts in **1–3** [10]. Compound **2** with non-peripheral glycerol substitutions had the highest bathochromic shift with shifts of 30–100 nm and the highest molar extinction values, which is a common phenomenon seen in phthalocyanines with non-peripheral substitutions [29], [30], [31]. Furthermore, non-peripheral substitutions also have the effect of enhancing monomerization of phthalocyanines which was evidenced in this study with the observation of the highest molar extinction coefficient values in **2** in all the solvents tested compared to the other compounds. This effect was found to be lowered in the case of **3** where one of its non-peripheral glycerol was replaced with a hydrophobic iodine atom. On their fluorescence properties, these phthalocyanines demonstrated very similar fluorescence emission wavelength at ∼700 nm with relatively low fluorescence quantum yields (Φ_F_) in DMSO ranging from 0.03 to 0.04 as calculated by using ZnPcS_4_ (Ф_F_ = 0.07 in DMSO) [13] as a reference. Glycerol substitution appeared to lower the fluorescence quantum yield of these phthalocyanines regardless of the heavy atom substitution.

**Table 2:** Photophysical properties of phthalocyanines **1**, **2** and **3** in relation to ZnPcS_4_.

Pc	DMSO		Ethanol		PBS		PBS+2% Triton x-100		φ_F_
	λ_max_	ε (M^−1^cm^−1^)	λ_max_	ε (M^−1^cm^−1^)	λ_max_	ε (M^−1^cm^−1^)	λ_max_	ε (M^−1^cm^−1^)	
ZnPcS_4_	676	114 000	666	27 000	606	23 000	606	20 000	0.07
**1**	680	180 000	674	137 000	632	22 000	682	56 000	0.04
**2**	704	196 000	698	190 000	654	58 000	704	179 000	0.03
**3**	698	155 000	690	112 000	646	25 000	698	142 000	0.03

Pc = Phthalocyanine; φ_F_ = fluorescence quantum yield in DMSO.

**Figure 3:**
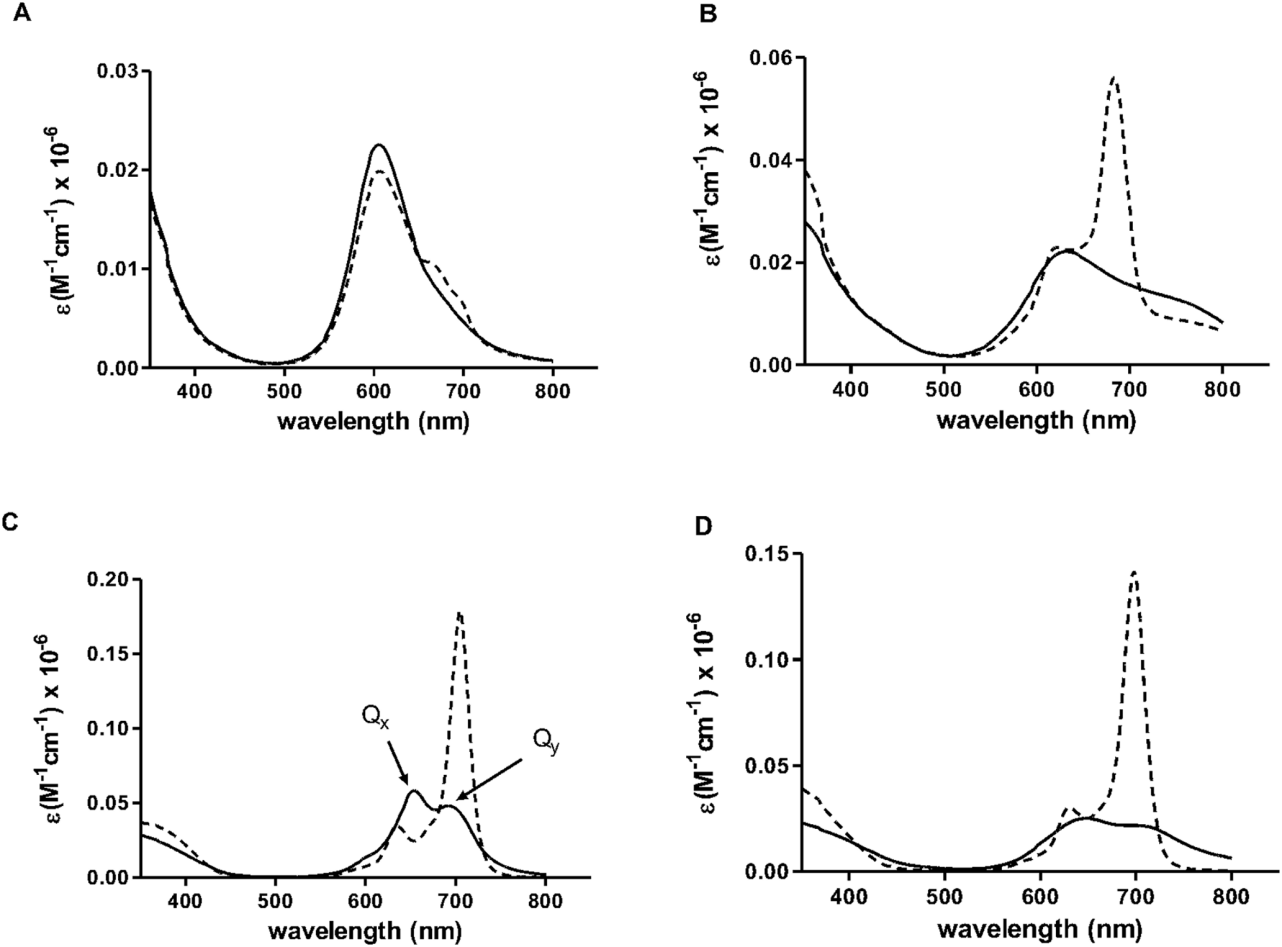
Absorption spectra of zinc (II) phthalocyanines in PBS with and without a surfactant. Absorption spectra of (A) ZnPcS_4_, (B) Phthalocyanine **1**, (C) Phthalocyanine **2**, and (D) Phthalocyanine **3**, in (**—**) PBS and (---) PBS+2% Triton X-100. Addition of a surfactant red-shifted the absorption maxima and resulted in higher molar extinction coefficients in **1**, **2** and **3** as shown by the increased intensity of Q_y_ band.

Aggregation of phthalocyanine is often associated with low molar extinction coefficient values. Consistently, all phthalocyanines here had the highest molar extinction coefficient values when measured in DMSO and the lowest when measured in PBS (Table 2). This suggests that phthalocyanines favour formation of monomers in DMSO while, in PBS they probably exist in aggregated forms. This is further supported by Figure 2, which shows that the excitation spectrum of each phthalocyanine in DMSO was similar to its corresponding absorption spectrum in the same solvent, indicating that all the phthalocyanines appeared in their monomeric forms in DMSO. This finding is consistent with other studies which demonstrated in case of aggregation, the excitation and absorption spectra are not overlapping [14], [32]. As additional proof, the aggregation of phthalocyanine analogs in PBS was reversed in the presence of a surfactant. This was indicated by an increase in the intensity of the Q_y_ band following the addition of 2% Triton X-100 as a surfactant (Table 2 & Figure 3). This phenomenon was observed for all phthalocyanine analogs, but not for ZnPcS_4_.

### Comparative Singlet Oxygen Generation Rate

**Figure 4:**
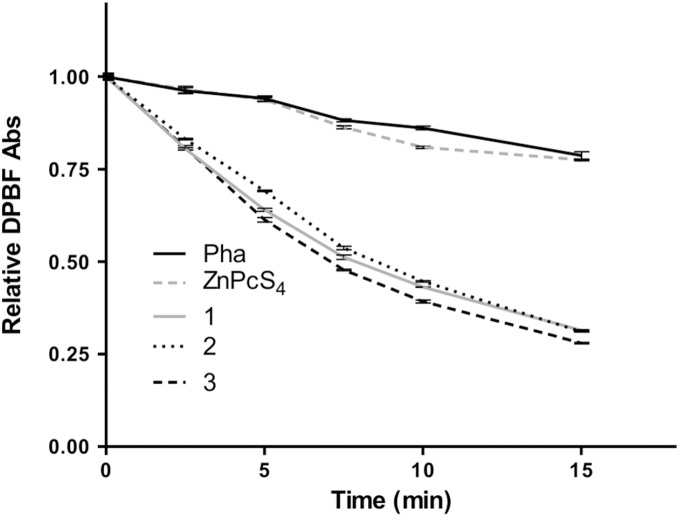
Comparative singlet oxygen generation rate of zinc (II) phthalocyanines. Comparative singlet oxygen generation of the phthalocyanines relative to pheophorbide-a (Pha) at 0.5 µM. The rate of reduction of DPBF absorbance caused by phthalocyanine **1**, **2** and **3** was higher in comparison with ZnPcS_4_ and Pha.

Singlet oxygen generation ability is one of the determining factors for PDT efficiency, as these highly reactive species is the primary cytotoxic agent responsible for photobiological activity. Comparative singlet oxygen generation rate of the phthalocyanines was determined at 0.5 µM in isopropanol with pheophorbide *a* as a reference, by monitoring the disappearance of 1,3-diphenylisobenzofuran (DPBF, a singlet oxygen quencher) absorbance (Figure 4). From Table 3, the singlet oxygen generation rates of phthalocyanine **1**, **2** and **3** were similar and were approximately 2.5 folds higher than ZnPcS_4_. This suggests that glycerol-substitution, regardless of the position of glycerol groups, enhanced the singlet oxygen generation ability of phthalocyanines.

**Table 3:** Log *P*, singlet oxygen generation rate and *in vitro* phototoxicity of phthalocyanines **1–3**.

Pc	log *P*	^1^O_2_ generation rate	Activity IC_50_ (µM)					
			MCF-7		HSC-2		HCT 116	
			0 J/cm^2^	4.0 J/cm^2^	0 J/cm^2^	4.0 J/cm^2^	0 J/cm^2^	4.0 J/cm^2^
ZnPcS_4_	−1.7±0.4	0.32±0.03	>100	>100	>100	>100	>100	>100
**1**	0.9±0.1	0.80±0.26	>100	>100	>100	>100	>100	>100
**2**	0.9±0.1	0.82±0.14	>100	2.9±0.8*	>100	2.8±0.9*	>100	3.2±0.2*
**3**	1.2±0.2	0.79±0.12	5.2±0.3*	0.06±0.02*	>100	0.06±0.02*	7.5±1.6*	0.04±0.01*

*In vitro* phototoxicity is given in the value of IC_50_ which is the concentration that inhibits 50% of cell viability. ^1^O_2_ generation rate is the singlet oxygen generation rate relative to Pha. The significance between different groups with ZnPcS_4_ was compared by one-way analysis of variance (ANOVA), where * indicates p<0.01.

The introduction of heavy atoms such as iodine and bromine to photosensitizers is able to enhance their singlet oxygen generation ability by improving the ISC and the triplet lifetime of the photosensitizers [15], [16], [33], [34], [35]. In this study, however, mono-iodo substitution in compound **3** did not result in higher singlet oxygen generation rate than compound **1** and **2**. This was probably due to the more severe aggregation problem caused by the hydrophobic nature of the iodine atom in **3**, as demonstrated by the lowest molar extinction coefficient value of compound **3** compared to others, which would have in turn lowered the singlet oxygen generation ability of **3**.

### Partition Coefficient (Log *P*)

Partition coefficient (log *P*) is defined as the ratio of concentrations of a dissolved substance in a two-phase system consisting of two immiscible solvents e.g. n-octanol and water at equilibrium. It reflects the level of hydrophilicity and hydrophobicity of a compound, which determines the permeability of the compound across the lipid bilayers of cell membrane. In this study, the log *P* values of the phthalocyanines were measured using the shake-flask method. Our findings showed that **3** was more lipophilic than the other phthalocyanines as it recorded a higher log *P* value. We also showed that the presence of glycerol groups improved the hydrophilicity of the phthalocyanines corresponding to the number of glycerol groups present (Table 3). Both phthalocyanines **1** and **2,** which were tetra-glycerol substituted, recorded lower log *P* values of 0.9 when compared to **3** which was tri-glycerol substituted, with a log *P* value of 1.3. While ZnPcS_4_, with tetra-sulfonation showed a much higher hydrophilicity with a log *P* value of −1.8.

### Cellular Uptake Study

**Figure 5:**
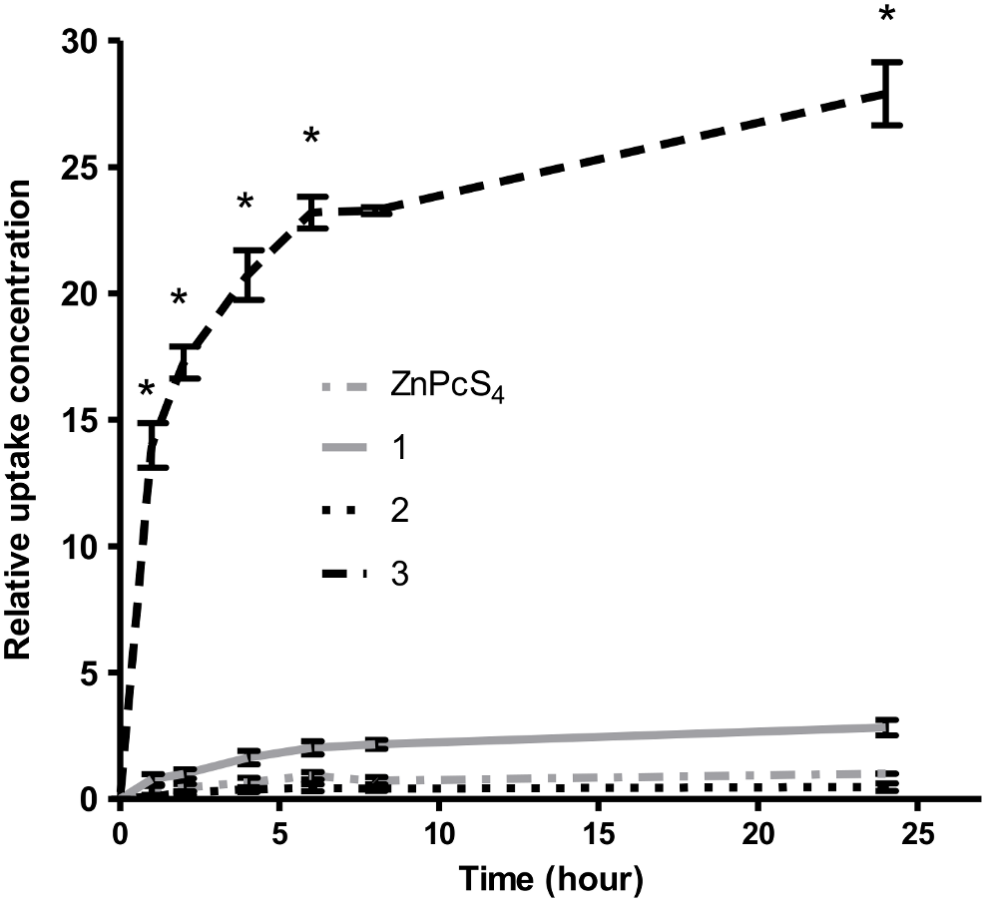
Cellular uptake profile of zinc (II) phthalocyanines. Cellular uptake of phthalocyanines **1**, **2** and **3** (concentration) relative to the uptake of ZnPcS_4_ when measured at 24 hours post treatment. **3** with tri-glycerol and mono-iodo non-peripheral substitution had the highest uptake by cells reaching about 30 fold (p<0.01) more than ZnPcS_4._ The uptake of ZnPcS_4_, **1** and **2** were relatively low and were not significantly different.

Based on the cellular uptake study performed (Figure 5), **3** was rapidly taken up by cells at the first 2 h of incubation with a 20-fold higher (p<0.01) amount than ZnPcS_4_ and reaching plateau with a 30-fold higher (p<0.01) amount than ZnPcS_4_ at 24 h. On the other hand, **1**, **2** and ZnPcS_4,_ being more hydrophilic, were poorly taken up by cells. The uptake of **1** and **2** were not statistically different from ZnPcS_4_.

Both hydrophilicity and lipophilicity play a part in determining the cellular uptake of a compound. Although it is generally assumed that lipophilic compounds have higher tendencies to cross the membrane lipid bilayers resulting in increased uptake of the compounds into cells, several studies have shown that uptake of certain porphyrins demonstrated nonlinear correlation with their lipophilicity parameter [36], [37]. The reason is that the extremely lipophilic porphyrins tend to form aggregates in media and thus prevent plasma membrane diffusion. On the other hand, a certain level of hydrophilicity is neccessary for effective delivery of photosensitizers into cells. Therefore, a balance of hydrophilic/lipophilic characteristic is usually required for optimum photosensitizer uptake by cells.

### *In vitro* PDT Activity

The *in vitro* phototoxicity of these phthalocyanines was determined on a panel of cancer cell lines, namely MCF-7, HCT-116 and HSC-2 with an irradiation fluence of 4.0 J/cm^2^. Data presented in Table 3 indicated that the *in vitro* phototoxicity potency of the phthalocyanines was ranked in the following order: phthalocyanine **3**> phthalocyanine **2**> phthalocyanine **1**≈ZnPcS_4._ The attachment of an iodo moiety in **3** resulted in a significant increase of 60-folds (p<001) in light-mediated cytotoxicity with IC_50_ values ranging 0.04–0.06 µM compared to **2** with IC_50_ values of 2.8–3.2 µM. Meanwhile, for **1** and ZnPcS_4_, there was no significant loss of cell viability for treatment concentrations of up to 100 µM. Only **3** showed dark toxicity in two of the cell lines tested, MCF-7 and HCT-116 with IC_50_ values of 5.2 µM and 7.5 µM respectively. The dark-light IC_50_ ratio of approximately 100-fold was statistically significant with p<0.01.

The lack of *in vitro* phototoxicity of ZnPcS_4_ is possibly caused by a combination of its relatively low singlet oxygen quantum yield and poor cellular uptake (Figure 5) as previously reported [38]. However for phthalocyanine **1**, despite having high singlet oxygen generation rate, the compound failed to exert *in vitro* PDT activity probably due to the formation of precipitates which was observed following addition of the compound in media. This occurrence was also reported for tetra-ethyleneglycol-substituted zinc (II) phthalocyanines whereby greater aggregation was observed in peripheral rather than non-peripheral conjugates [12], [32], [39]. On the other hand, phthalocyanine **2**, a positional isomer of **1**, did not precipitate when added in culture media and exerted better phototoxicity compared to **1**, indicating that substitution at non-peripheral position is preferable for good phototoxicity. These observations are consistent with previous experiments performed by our group against HT-29 cells [11].

In present study, the photosensitizer was neither removed nor the cells were rinsed before illumination was performed. This would be closer to the physiological condition, as there would be photosensitizer accumulating at the interstitial space between the tumor cells in actual condition, similar to photosensitizer that are still present in the media. Even though **3** has similar singlet oxygen generation rate compared to **2**, the enhanced potency exhibited by **3** could be attributed mainly to its abundant cellular uptake and partly to its inherent dark toxicity. As shown in the cellular uptake data, the amount of **3** taken up by cells was 20-fold higher (p<0.01) than the other phthalocyanines which would have contributed to the highest phototoxicity observed among the compounds. The lack of photodynamic efficacy of free photosensitizer was probably due to the high reactivity and short lifetime of singlet oxygen generated which was rapidly annihilated by organic components presence in the culture media.

### PDT-induced Vascular Occlusion in CAM Model

As PDT also acts by disrupting the blood vessels feeding the tumor, the photodynamic efficiency of these phthalocyanines in causing vasculature occlusion was evaluated using the CAM model. In order to introduce a hydrophobic compound into the CAM systemic circulation, a suitable dosing vehicle was first developed to ensure complete solubility of the compounds. Using **3** as representative, formulation was prepared with commonly used surfactants such as PEG 400 and CrEL. The safety profiles of **3** and its vehicle in terms of chick embryos survival were examined 24 h following administration. Results tabulated in Table 4 showed that when formulated in saline: PEG400: EtOH (5∶3∶2), compound **3** failed to achieve desirable solubility. Formulation using 5% DMSO in 10% glucose resulted in precipitation of compound **3** upon dosing and caused a decrease in embryo survival rate. On the other hand, formulation of **3** with 5% CrEL, 5% EtOH in saline appeared to be the most appropriate as the compound was able to solubilize at sufficient concentrations and no precipitation was observed upon injection into the CAM vasculature. Furthermore, the formulation was well-tolerated by the embryos and did not cause vasculature disruption or reduce embryo survival. This combination of CrEL and EtOH has been successfully used as excipients in other photosensitizer studies for PDT efficacy in CAM model [19], [40]. Based on this and the findings here, this formulation was also used on phthalocyanines **1**, **2** and ZnPcS_4_ for subsequent vasculature occlusion studies.

**Table 4:** The effect of intravenous administration of phthalocyanine **3** formulation on chick embryo survival.

Formulation	Preparation for 0.5 mM Pc 3	Pc 3 (nmol/embryo)	Precipitationupon dosing	Embryo 24- hour survival
Saline: PEG400: EtOH (5∶3∶2)	0.325 mM (from abs measurement)	6.5	No	9/10
5% CrEL: 5% EtOH in saline	5 mM of **3** in 50% CrEL: 50% EtOH.Dilute with saline.	10	No	9/10
5% DMSO in 10% glucose	10 mM of **3** in DMSO.Dilute with 10% glucose.	10	Yes (sometimes)	3/8

**Figure 6:**
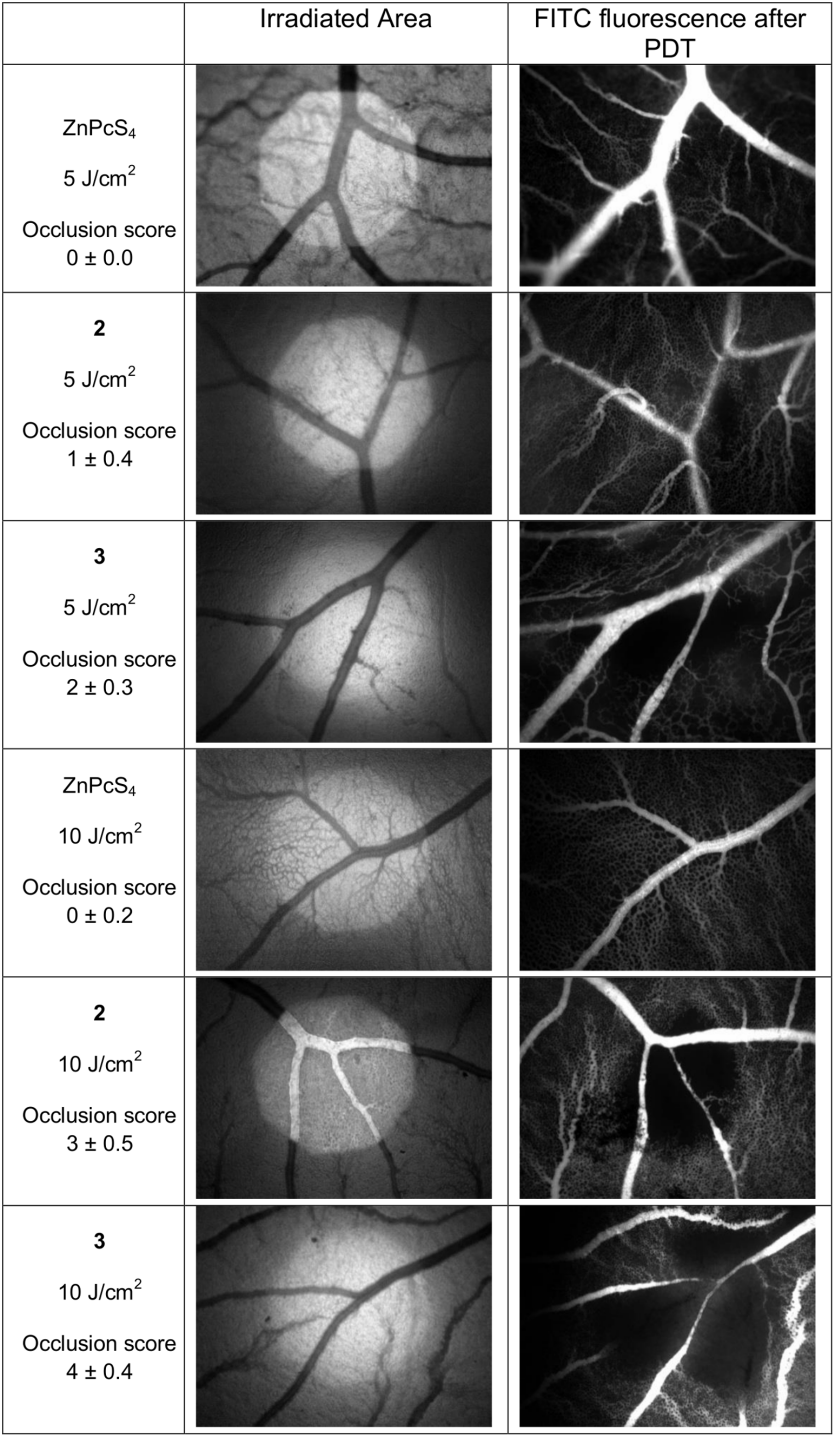
Vasculature damage on CAM models. Representative angiographies of the CAM blood vessels before and after PDT, illustrating the vascular occlusion efficacy induced by standard (ZnPcS_4_), phthalocyanines **2** and **3** at 10 nmol/embryo. Irradiation was performed at 600–800 nm of excitation wavelength, with light doses of 5 J/cm^2^ and 10 J/cm^2^. Objective magnification was 4×. Occlusion score represents the mean ± SEM of ten embryos for each treatment group. At the lower light dose of 5 J/cm^2^, **2** was able to cause partial closure of the capillary system and **3** was able to cause complete closure of the capillary system. At the higher light dose of 10 J/cm^2^, **2** had the ability to close up vessels of diameter of <30 µm, whereas **3** caused total closure of vessels of <70 µm in diameter.

The drug was intravenously injected and irradiation was performed at 600–800 nm of excitation wavelength, with a light dose of 5 or 10 J/cm^2^. The extent of vasculature occlusion was scored immediately after treatment according to Table 1 and the occlusion scores were expressed as mean ± SEM of ten embryos per group (Figure 6). From the experiment, PDT treatment of **2** and **3** at 10 nmol/embryo and light irradiation of 5 J/cm^2^ was able to induce partial closure of CAM capillaries with diameter <10 µm and blood vessels with diameter <30 µm respectively. In contrast, ZnPcS_4_ at the same drug and light dose failed to demonstrate vascular occlusion effects. A higher occlusion level was achieved when the light dose was increase to 10 J/cm^2^ with **2** and **3** inducing total occlusion of vessels with diameter <30 µm and <70 µm, respectively at 10 nmol/embryo. Phthalocyanine **1** was observed to form precipitation in the blood vessel immediately after injection and was not studied further. Our findings suggest that **2** and **3** are potentially useful candidates as PDT agents, because they are able to cause vasculature damage in CAM model with better potency than ZnPcS_4_. Although the desirable photodynamic effect of complete closure of blood vessels at the irradiation site was not achieved here, we deduce that it could be achieved by increasing either the drug or the light dose. The observed higher vasculature occlusion efficiency of **3** compared to **2** correlates with the data obtained in the *in vitro* phototoxicity study.

## Conclusions

In this study, the photodynamic efficacies of zinc (II) phthalocyanines following various modes of glycerol-substitution were investigated. The introduction of multiple glycerol groups at different positions and also with the combination of one iodine atom have resulted in stronger and red shifted absorbance compared to the reference tetrasulfonated zinc (II) phthalocyanine. These modifications also improved their singlet oxygen production. However, peripheral substitution in phthalocyanine **1** formed aggregation at a higher degree than non-peripheral substitution, resulting in a loss of potency and limiting its investigations in PDT efficacy studies in CAM. Meanwhile, both the non-peripherally substituted phthalocyanine **2** and mono-iodo tri-glycerol substituted phthalocyanine **3** showed potent *in vitro* phototoxicity and ability to induce vasculature damage in the CAM model. This is attributed to the aggregation lowering effect of the non-peripheral substitution pattern, and the enhanced cellular uptake of the more lipophilic compound **3** which contains one iodine atom. Although phthalocyanine **3** exhibited slight *in vitro* dark toxicity, the dark-light IC_50_ ratio (approximately 100∼fold) is sufficient to allow the compound to occlude blood vasculature without causing toxicity to the chick embryo. In conclusion, data obtained from this study suggest that phthalocyanines **2** and **3** have the potential to be further explored as clinically useful agents for PDT of cancer and will therefore be further investigated in *in vivo* tumor models.
